# Comprehensive Identification and Description of Mitochondrial DNA Haplogroups and Sub-Haplogroups in Snow Sheep (*Ovis nivicola*) in Support of Their Evolutionary History and Conservation

**DOI:** 10.3390/ani16193018

**Published:** 2026-09-25

**Authors:** Arsen V. Dotsev, Innokentiy M. Okhlopkov, Olga A. Koshkina, Mikhail G. Bondar, Dennis I. Litovka, Andrey A. Sitsko, Taras P. Sipko, Neckruz F. Bakoev, Nikolai V. Mamaev, Veronika R. Kharzinova, Tatiana E. Deniskova, Maria N. Semerikova, Vugar A. Bagirov, Michael N. Romanov, Darren K. Griffin, Ivica Medugorac, Gottfried Brem, Natalia A. Zinovieva

**Affiliations:** 1 L. K. Ernst Federal Research Center for Animal Husbandry, Dubrovitsy, 142132 Podolsk Urban Okrug, Russia; olechka1808@list.ru (O.A.K.); nekruz82@bk.ru (N.F.B.); veronika0784@mail.ru (V.R.K.); horarka@yandex.ru (T.E.D.); vugarbagirov@mail.ru (V.A.B.); n_zinovieva@mail.ru (N.A.Z.); 2 Institute of Biological Problems of the Cryolithozone, Siberian Branch of the Russian Academy of Sciences, 677007 Yakutsk, Russia; imo-ibpc@yandex.ru (I.M.O.); mamaev_88@bk.ru (N.V.M.); 3 Joint Directorate of Taimyr Reserves, 663305 Norilsk, Russia; mikisayan@yandex.ru; 4 Chukotka Arctic Research Center, 689000 Anadyr, Russia; d-litovka@yandex.ru (D.I.L.); koshornikovamn@mail.ru (M.N.S.); 5 Rosokhotrybolovsoyuz Association, 125212 Moscow, Russia; sitsko@mnr.gov.ru; 6 A. N. Severtsov Institute of Ecology and Evolution, Russian Academy of Sciences, 119071 Moscow, Russia; sipkotp@mail.ru; 7 School of Natural Sciences, University of Kent, Kent, Canterbury CT2 7NJ, UK; d.k.griffin@kent.ac.uk; 8 Animal Genomics and Bioresource Research Unit (AGB Research Unit), Faculty of Science, Kasetsart University, Chatuchak, Bangkok 10900, Thailand; 9 Preimplantation Genetics Group, EGA Institute for Women’s Health, University College London, London WC1E 6HX, UK; 10 Population Genomics Group, Department of Veterinary Sciences, LMU Munich, 80539 Munich, Germany; ivica.medjugorac@gen.vetmed.uni-muenchen.de; 11 Institute of Animal Breeding and Genetics, University of Veterinary Medicine, 1210 Vienna, Austria; gottfried.brem@vetmeduni.ac.at

**Keywords:** snow sheep (*Ovis nivicola*), mitochondrial DNA, mitochondrial genome, haplogroups, phylogeography, population genetics, genetic diversity, evolutionary history, Siberia, Russian Far East, Pleistocene refugia, conservation

## Abstract

Mitochondrial DNA is a powerful marker for reconstructing maternal lineages and phylogeographic history in wild species. The snow sheep (*Ovis nivicola*), a mountain ungulate endemic to Siberia and the Russian Far East, is characterized by strong geographic fragmentation and a complex taxonomic structure. A formal mitochondrial haplogroup classification system for the species has, however, been lacking. In this study, we sequenced and analyzed 257 complete mitochondrial genomes representing all major populations across the species’ range. Using phylogenetic reconstruction and diagnostic mutations (present in 100% of individuals within clades), we identified four major haplogroups (A–D) and multiple nested sub-haplogroups with clear geographic structuring. Divergence times were estimated using Bayesian analysis in BEAST 2.7.7 with a relaxed lognormal clock model, calibrated by the divergence between *O. dalli* and *O. canadensis*. The results showed that haplogroup A is almost exclusive to Kamchatka, haplogroup B is predominant in northern populations, haplogroup C is the most widespread and diverse across the species range, and haplogroup D is largely restricted to the Verkhoyansk region. The mitochondrial data largely support traditional subspecies taxonomy but reveal mitochondrial polyphyly in some subspecies, particularly *O. n. lydekkeri* and *O. n. koriakorum*. This study provides the first comprehensive mitochondrial haplogroup system for snow sheep and shows that the observed mitochondrial phylogeographic patterns are consistent with an important influence of Pleistocene glacial cycles and prolonged geographic isolation on its current genetic structure. These findings offer important insights for conservation management and for understanding the evolutionary history of wild sheep in Northeast Asia.

## Simple Summary

Snow sheep (*Ovis nivicola*) are wild animals inhabiting remote and isolated mountain ranges across Siberia and the Russian Far East. Because of strong geographic isolation, different populations have accumulated distinct genetic differences over time. Until now, a comprehensive understanding of their maternal genetic lineages was lacking. In this study, we analyzed complete mitochondrial DNA genomes from 257 snow sheep collected across the entire species range. We identified four major maternal lineages (we called these haplogroups A, B, C, and D) and numerous sublineages within them, each associated with specific geographic regions. For example, haplogroup A is almost exclusively found in Kamchatka, whereas haplogroup C is the most widespread. Some traditionally recognized subspecies were shown to contain multiple maternal lineages, indicating that they are genetically more complex than previously thought. Our results suggest that snow sheep survived the climatic fluctuations of the Ice Age in separate mountain refugia and subsequently expanded from these isolated areas. This new mitochondrial haplogroup classification system will support more effective conservation strategies, help define appropriate management units, and improve our understanding of how large mammals persisted across Siberia and the Russian Far East during periods of dramatic climate change.

## 1. Introduction

One of the most effective approaches for investigating the evolutionary and demographic history of species is the analysis of mitochondrial DNA (mtDNA), which is maternally inherited and does not undergo genetic recombination. Due to its relatively high mutation rate and stable inheritance pattern, mtDNA is widely used in phylogenetic and phylogeographic studies to identify historically isolated maternal lineages and reconstruct patterns of population divergence, migration, and demographic change [1,2,3].

A mitochondrial haplogroup represents a group of individuals sharing a common set of diagnostic mtDNA mutations inherited from a common maternal ancestor [1,2]. A haplotype, in contrast, refers to a specific mitochondrial DNA sequence variant—a particular combination of mutations observed in an individual or a set of individuals—so that a haplogroup comprises a group of related haplotypes. Haplogroup analysis provides a powerful framework for describing intraspecific genetic structure and tracing the evolutionary history of populations. In wild animals, mitochondrial haplogroups are particularly informative for identifying phylogeographic lineages, reconstructing historical refugia and postglacial dispersal routes. They also can be employed to evaluate the effects of long-term geographic isolation [3,4,5].

Characterization of mitochondrial haplogroups is important for conservation genetics, as deeply divergent or geographically restricted maternal lineages may indicate unique evolutionary histories and population distinctiveness. High haplotype and haplogroup diversity often signals long-term persistence, historical admixture, or survival in multiple refugia, whereas low diversity may reflect population bottlenecks or prolonged isolation [4,6]. Therefore, establishing a comprehensive mitochondrial haplogroup system can provide valuable information for the conservation and management of genetically structured populations.

The most comprehensive mitochondrial haplogroup classification system has been developed for humans, where hierarchical mtDNA nomenclature is widely used to reconstruct evolutionary history, migration patterns, and demographic processes [7]. Similar approaches have subsequently been applied to numerous domestic animal species, including cattle, pigs, goats, chickens, horses and sheep [8,9,10,11,12,13,14]. In these species, mitochondrial haplogroups are defined by lineage-specific sets of mutations and have proven instrumental in studying domestication history, phylogeographic structure and historical admixture.

In some cases, mitochondrial haplogroup systems established for domestic species can also be applied to their wild ancestral forms. For example, the haplogroup classification developed for domestic goats is suitable for bezoar ibex (*Capra aegagrus*) [8], and the domestic sheep system can successfully be applied to Asian mouflon (*Ovis gmelini*) and European mouflon (*O. aries musimon*) [9]. However, this haplogroup system is not directly applicable to other representatives of the genus *Ovis*, including snow sheep (*O. nivicola*) [15,16].

*O. nivicola* is a wild mountain sheep distributed across Siberia and the Russian Far East, inhabiting isolated mountain systems from the Putorana Plateau and Verkhoyansk Range to Chukotka and the Kamchatka Peninsula [17,18]. The species is characterized by pronounced geographic fragmentation, with many populations separated by extensive lowland areas that likely limit gene flow. Several geographically isolated forms or subspecies have been proposed based on morphological and genetic data [19,20,21]. Previous molecular studies revealed substantial population structure and deep genetic divergence, suggesting a complex evolutionary history shaped by long-term isolation and repeated Pleistocene climatic fluctuations [16,22,23,24]. Representative individuals of *O. nivicola* from the Putorana Plateau population are shown in Figure 1.

Although mitochondrial phylogeographic studies have been conducted for many wild mammal species, formalized haplogroup classification systems covering the entire species range remain rare outside humans and domestic animals [25]. Most studies describe regional mitochondrial lineages or clades without establishing a unified hierarchical nomenclature supported by diagnostic mutations [26,27,28]. Therefore, the development of a comprehensive mitochondrial haplogroup system for *O. nivicola* represents an important step toward standardizing the description of maternal genetic diversity and advancing our understanding of its evolutionary and phylogeographic history.

The aim of the present study was therefore to identify and characterize the major mitochondrial haplogroups and sub-haplogroups of snow sheep across their entire species distribution range using complete mitochondrial genome sequences.

## 2. Materials and Methods

### 2.1. Sample Collection and DNA Sequencing

Muscle tissue samples of snow sheep (*O. nivicola*) were collected from across all major parts of the species’ distribution range, ensuring representation of the principal geographic populations. Sampling covered key mountain systems throughout Siberia and the Russian Far East.

Samples were obtained from multiple sources. A portion was collected during dedicated field expeditions. Additional material was provided by licensed trophy hunters and by local indigenous residents, who are legally permitted to harvest snow sheep under regional quota systems. For populations of conservation concern (including those listed in regional or national Red Data Books), samples were obtained opportunistically from animals that had died of natural causes, including remains of individuals killed by predators, as well as from museum collections. No samples were collected from live animals, and no animals were killed specifically for this study.

In total, 257 samples were included in the analysis, covering all major geographic regions across the species’ range, including Kamchatka Peninsula mountains, Koryak Mountains, Chukotka, northeastern Yakutia, Okhotsk region, and Putorana Plateau. Detailed information on sampling localities, population assignment, and sample sizes is provided in Table 1.

Total genomic DNA was extracted from muscle tissue samples using a magnetic bead-based purification method following the manufacturer’s protocol. DNA concentration and quality were assessed using a Qubit fluorometer (Thermo Fisher Scientific, Waltham, MA, USA).

Library preparation was performed using the TruSeq DNA kit (Illumina). Sequencing was carried out in 150-bp paired-end mode on Illumina platforms. Mitochondrial-targeted libraries were sequenced on a MiSeq system, while whole-genome sequencing (WGS) libraries were sequenced on a HiSeq 2500 system. In total, 170 samples were obtained through a mitochondrial-targeted approach and 103 samples via WGS, with partial overlap between the two datasets. Comparison of the overlapping samples confirmed that the mitochondrial sequences generated by the two approaches were fully concordant. Mitochondrial sequences from WGS data were extracted in silico and processed using the same alignment, variant-calling and consensus-generation pipeline as the mitochondrial-targeted samples. The average sequencing depth of the mitochondrial genome was 1308 ± 57× for the mitochondrial-targeted dataset and 10,450 ± 952× for the WGS dataset (calculated with samtools depth).

### 2.2. Sequence Assembly and Alignment

Raw sequencing reads were subjected to quality control and adapter trimming using fastp (version 0.24.0; [29]). Adapter detection for paired-end reads, overrepresentation analysis, base correction, and right-end quality trimming were enabled using the —detect_adapter_for_pe, —overrepresentation_analysis, —correction, and —cut_right options, respectively. Cleaned reads were aligned to the reference mitochondrial genome of *O. nivicola* (NCBI Reference Sequence: NC_039431.1; 16,471 bp; [15]) using bwa-mem2 (version 2.2.1; [30]) with default parameters.

The resulting alignments were processed using SAMtools 1.22.1 [31], including sorting, mate-pair fixing, and duplicate marking. Indexed BAM files were generated for downstream analyses.

Variant calling was performed using BCFtools 1.22 [32] with the mpileup and call functions under a haploid model appropriate for mitochondrial genomes (—ploidy 1). Variant normalization and filtering were conducted using BCFtools norm and BCFtools filter to remove low-quality variants.

Consensus mitochondrial genome sequences were reconstructed for each sample using BCFtools consensus with the filtering expression QUAL > 50 & MIN(DP) > 30 (sites not meeting these thresholds were masked as N). Final consensus sequences were exported in FASTA format. Mitochondrial genome sequences were aligned using MUSCLE (version 3.8; [33]) with default parameters.

### 2.3. Haplogroup Definition and Diagnostic SNP Identification

Samples were assigned to haplogroups based on clustering patterns inferred from sequence similarity and phylogenetic relationships. Initial grouping was refined by identifying shared diagnostic mutations across individuals. Haplogroups were hierarchically structured (e.g., A, A1, A2a, B2b, C1a, etc.), reflecting nested phylogenetic relationships within *O. nivicola* mitochondrial diversity.

To identify haplogroup-specific polymorphisms, a custom script written in R (version 4.3.2; [34]) was used. All candidate diagnostic mutations identified computationally were subsequently validated by manual inspection of the multiple sequence alignment. For each nucleotide position, the frequency of each variant was calculated within and across haplogroups. Diagnostic SNPs were defined as those present in 100% of individuals within a given haplogroup or sub-haplogroup and completely absent (0%) in all individuals outside that group. Only mutations meeting this strict specificity threshold were retained as diagnostic markers. Only SNPs were considered; insertions and deletions (indels) were excluded from the analysis.

### 2.4. Phylogenetic Analysis and Divergence Time Estimation

One representative sample from each major haplogroup and sub-haplogroup (including nested subclades) was selected for phylogenetic reconstruction and divergence time estimation. Mitochondrial genomes were annotated using MITOS2 (version 2.1.10; [35]) on the Galaxy platform [36,37]. A concatenated alignment consisting of 13 protein-coding genes and two ribosomal RNA genes (12S and 16S rRNA) was used for subsequent analyses.

A median-joining network [38] to visualize haplotype relationships among all 257 individuals was constructed in PopART (version 1.7; [39]).

The best-fit partitioning schemes and nucleotide substitution models for each locus were determined using PartitionFinder2 [40]. The resulting partition scheme and best-fit substitution models are provided in Supplementary Table S1. Bayesian phylogenetic inference and divergence time estimation were performed in BEAST (version 2.7.7; [41]). A relaxed lognormal clock model and a Birth-Death tree prior were applied. The analysis was run for 100,000,000 generations, with sampling every 10,000 generations and a burn-in of 10% (10,000,000 generations). *O. dalli* (NCBI Accession No. NC_039432; [15]) and *O. canadensis* (NCBI Accession No. MH094035; [42]) were used as outgroups. The divergence time between *O. dalli* and *O. canadensis* of approximately 950 kya, as estimated by Rezaei et al. [43], was used as a fixed calibration point.

Convergence of the runs was assessed in Tracer (version 1.7.2; [44]), ensuring effective sample size (ESS) values exceeded 200 for all key parameters. The maximum clade credibility tree with median node heights was generated using TreeAnnotator (version 2.7.7; distributed with BEAST, version 2.7.7) and visualized using FigTree (version 1.4.2; [45]).

The geographic distribution of haplogroups was mapped in R (version 4.3.2) using the maps package (version 3.4.3) [46].

## 3. Results and Discussion

### 3.1. Phylogenetic Structure and Definition of Haplogroups

The assembled mitochondrial genomes ranged from 16,453 to 16,471 bp in length. After alignment to the reference sequence (NC_039431.1) and exclusion of sites with gaps or missing data, the final dataset comprised 16,437 positions. A total of 138 distinct haplotypes and 668 segregating sites were identified.

Analysis of the 257 complete mitochondrial genome sequences of *O. nivicola* revealed a clear hierarchical phylogenetic structure within the species. Based on patterns of shared diagnostic mutations and the median-joining network (Figure 2), four major mitochondrial haplogroups were identified and designated A, B, C, and D. Within each major haplogroup, multiple nested sub-haplogroups were resolved, reflecting pronounced phylogeographic structuring of snow sheep populations across Siberia and the Russian Far East.

To facilitate visualization and interpretation of phylogenetic relationships among lineages, a simplified Bayesian tree containing one representative haplotype from each haplogroup and sub-haplogroup was constructed and is presented in Figure 3. Divergence time estimates indicated deep separations among the major mitochondrial lineages, particularly between haplogroup A and the remaining clades.

The geographic composition of the identified mitochondrial haplogroups and sub-haplogroups is summarized in Table 2.

Analysis of mitochondrial genome variation identified multiple diagnostic nucleotide positions specific to individual haplogroups and sub-haplogroups. Using the criteria described in Section 2.3, we identified sets of haplogroup- and sub-haplogroup-specific SNPs distributed across the mitochondrial genome. The complete list of diagnostic mutations, their genomic positions relative to the reference sequence, and specificity values is provided in Supplementary Table S2. Each major haplogroup and its constituent sub-haplogroups were characterized by multiple lineage-specific mutations, with the number of diagnostic positions ranging from a few to several tens depending on the phylogenetic depth and distinctiveness of the clade (Supplementary Table S3).

The geographic distribution of the identified mitochondrial haplogroups and sub-haplogroups across the species range is shown in Figure 4.

### 3.2. Geographic Distribution and Composition of Haplogroup A

Haplogroup A was the most divergent mitochondrial lineage identified within *O. nivicola*. Phylogenetic reconstruction indicated that this lineage separated from all other extant snow sheep haplogroups approximately 640 kya (95% HPD: 490–790 kya), suggesting a long period of historical isolation.

A total of 73 diagnostic positions across the complete mitochondrial genome distinguished all individuals belonging to haplogroup A from the remaining snow sheep populations. These mutations were distributed across both coding and non-coding regions and showed high lineage specificity.

Geographically, haplogroup A was largely restricted to the Kamchatka Peninsula and a small subset of individuals from the Koryak Mountains. All sampled individuals from southern parts of the Sredinny Ridge clustered within this lineage, together with most samples from northern Kamchatka and four individuals from the Koryak population (4 of 31 analyzed samples).

Within haplogroup A, two major sub-haplogroups were identified. Sub-haplogroup A1, defined by 14 unique diagnostic substitutions, included all individuals from the southern Sredinny Ridge and one individual from the northern part of the ridge. Sub-haplogroup A2, characterized also by 14 lineage-specific mutations, comprised the remaining northern Kamchatka samples together with the Koryak individuals. Additional structuring within A2 gave rise to sublineages A2a, A2b, and A2c.

The strong geographic confinement and deep phylogenetic separation of haplogroup A indicate prolonged historical isolation of Kamchatkan populations. This pattern is consistent with previous findings based on nuclear genomic data [23].

### 3.3. Geographic Distribution and Composition of Haplogroup B

Haplogroup B was primarily associated with northern snow sheep populations, including the Putorana, Kharaulakh, and Selennyakh groups. In addition, six individuals from the Suntar-Khayata Range belonged to this lineage.

Phylogenetic analysis showed that haplogroup B diverged from the remaining mitochondrial lineages approximately 340 kya (95% HPD: 270–430 kya). This haplogroup was distinguished by 11 diagnostic nucleotide positions.

Within haplogroup B, two major sub-haplogroups were identified. Sub-haplogroup B1, defined by 23 unique diagnostic substitutions, consisted exclusively of individuals from the Putorana Plateau, forming a distinct regional lineage. Sub-haplogroup B2 (15 diagnostic substitutions) was further divided into B2a and B2b. Sub-haplogroup B2a included individuals from the Kharaulakh Range and most samples from the Orulgan Range, while B2b comprised individuals from the Selennyakh Range and all haplogroup B samples from the Suntar-Khayata Range.

The phylogeographic pattern of haplogroup B is particularly notable. This lineage occurs predominantly in geographically isolated northern populations that currently lack direct connectivity. At the same time, only a small number of individuals belonging to haplogroup B were detected in more southern regions. This distribution suggests that haplogroup B was historically more widespread across northeastern Siberia prior to the Late Pleistocene glaciations.

One possible explanation is that northern refugial areas located near the Arctic coastal regions enabled the long-term persistence of this mitochondrial lineage during glacial periods, while populations carrying haplogroup B disappeared from other parts of the species’ range. Subsequent postglacial expansion and recolonization by other snow sheep lineages likely contributed to the current fragmented distribution of haplogroup B.

### 3.4. Geographic Distribution and Composition of Haplogroup C

Haplogroup C was the most widespread and phylogenetically diverse mitochondrial lineage identified in *O. nivicola*. This haplogroup was distinguished from all other lineages by seven diagnostic nucleotide positions and occurred across a broad geographic area, extending from the Kodar and Stanovoy ranges in the west to Chukotka and the Koryak Mountains in the east.

Phylogenetic reconstruction indicated that haplogroup C diverged from haplogroup D approximately 290 kya (95% HPD: 220–360 kya). Within haplogroup C, five major sub-haplogroups (C1–C5) were identified, several of which exhibited further internal subdivision.

Sub-haplogroup C1 was defined by 10 diagnostic positions and divided into two lineages. C1a included individuals from the Suntar-Khayata Range (*n* = 5) and the Kolyma Mountains (*n* = 2), whereas C1b contained three individuals from the Moma Range.

Sub-haplogroup C2 was characterized by four diagnostic positions and comprised two major branches. C2a included samples from the Suntar-Khayata Range (*n* = 4) and the Chersky Range (*n* = 2). The second branch, C2b, was represented by seven individuals from the Chersky Range and one sample from the Selennyakh Range.

Sub-haplogroup C3 was defined by six diagnostic positions and also showed internal structuring. C3a was a relatively widespread lineage containing individuals from the Suntar-Khayata Range (*n* = 15), Stanovoy Range (*n* = 5), and Kolyma Mountains (*n* = 1). In contrast, C3b included two individuals from Suntar-Khayata and one individual from the Chersky Range.

Sub-haplogroup C4 was characterized by five diagnostic positions and represented the most geographically extensive and numerically dominant lineage within haplogroup C. It consisted of three sublineages. C4a included individuals from the Moma Range (*n* = 9) and Chersky Range (*n* = 7). C4b comprised samples from Kolyma Mountains (*n* = 8), Chukotka Mountains (*n* = 5), and Chersky Range (*n* = 2). C4c was dominated by individuals from the Koryak Mountains (*n* = 27), but also included two samples from Kolyma Mountains and one sample from Chukotka Mountains.

Finally, a provisional sub-haplogroup C5 was identified based on 21 diagnostic positions and was represented by a single individual from the Kodar Mountains. This individual formed a highly distinct mitochondrial lineage within haplogroup C. Recognition of C5 as a provisional sub-haplogroup is supported by the previously reported genetic characteristics of the Kodar population, which is small, geographically isolated and characterised by very low nuclear genetic diversity [24,47]. Nevertheless, additional sampling will be required to confirm the stability and population-level specificity of the diagnostic positions defining C5.

The broad geographic distribution and substantial internal diversity of haplogroup C suggest that this lineage was less affected by local extinction events during Pleistocene glacial cycles compared with other snow sheep haplogroups. Its persistence across multiple mountain systems and the presence of numerous geographically structured sublineages suggest long-term demographic stability and survival in several regional refugia. As a result, haplogroup C appears to have retained a large proportion of the ancestral mitochondrial diversity of snow sheep throughout the species’ range.

### 3.5. Geographic Distribution and Composition of Haplogroup D

Haplogroup D was restricted to individuals from the central part of the Verkhoyansk Range and a subset of samples from the Orulgan Range. This lineage was distinguished from all other *O. nivicola* haplogroups by 23 diagnostic nucleotide positions.

Within haplogroup D, two sub-haplogroups were identified. Sub-haplogroup D1 was defined by two diagnostic positions and consisted exclusively of individuals from the central Verkhoyansk population (*n* = 21). Sub-haplogroup D2 was characterized by 10 diagnostic positions and included additional individuals from the Verkhoyansk Range (*n* = 11) together with six samples from the Orulgan Range.

Compared with the other major mitochondrial lineages, haplogroup D exhibited a relatively restricted geographic distribution and limited internal diversity. Its confinement mainly to the Verkhoyansk region suggests long-term regional isolation and comparatively limited historical dispersal.

Notably, the occurrence of haplogroup D in the Orulgan population is consistent with previous evidence indicating a complex genetic structure of this group. Earlier nuclear genomic analyses suggested that the Orulgan population represents a genetically mixed or hybrid population positioned between neighboring snow sheep groups [22,24]. The co-occurrence of both haplogroup B and D mitochondrial lineages within Orulgan individuals further supports this interpretation and points to a history of secondary contact between distinct maternal lineages.

In contrast to the widely distributed haplogroup C, haplogroup D appears to represent a more localized mitochondrial lineage that likely persisted within a restricted refugial area during the Late Pleistocene climatic fluctuations.

### 3.6. Mitochondrial Haplogroups and Taxonomic Structure of Snow Sheep

The mitochondrial haplogroup system developed in this study is largely consistent with the traditional subspecies taxonomy of *O. nivicola*, while also revealing additional complexity and important refinements. All individuals of the Kamchatkan snow sheep (*O. n. nivicola*) belonged to haplogroup A, which was almost exclusively found in this subspecies, thereby supporting its strong genetic distinctiveness. The Koryak snow sheep (*O. n. koriakorum*) exhibited a mixed composition: the majority (~87%) carried haplogroup C, while approximately 13% possessed haplogroup A, consistent with a limited northward expansion of the Kamchatkan maternal lineage into the Koryak Mountains.

Haplogroup C was also dominant in the Okhotsk (*O. n. alleni*), Stanovoy (*O. n. potanini*), and Kodar (*O. n. kodarensis*) subspecies, as well as in most populations traditionally assigned to the Yakutian snow sheep (*O. n. lydekkeri*) from the Suntar-Khayata, Moma, and Chersky Ranges. In contrast, the Yakutian subspecies was mitochondrially heterogeneous, with individuals from the central Verkhoyansk Range belonging to haplogroup D.

Populations from the Kharaulakh Range, Selennyakh Range, and the Putorana Plateau (*O. n. borealis*) were united by haplogroup B. The clear affiliation of the Kharaulakh population with haplogroup B supports its genetic distinctiveness and the earlier proposal to recognize it as a separate subspecies (*O. n. ernsti*) [16,22,24].

Overall, the mitochondrial data largely confirm the validity of most currently recognized subspecies, but demonstrate that some of them, particularly *O. n. lydekkeri* and *O. n. koriakorum*, are mitochondrially polyphyletic. The observed distribution of haplogroups suggests that the evolutionary history of snow sheep was shaped by repeated Pleistocene glacial-interglacial cycles, involving long-term isolation in mountain refugia followed by limited postglacial expansions and secondary contacts.

Thus, the mitochondrial haplogroup system developed here provides a valuable complement to traditional morphological and nuclear genomic taxonomy, offering new insights into the evolutionary history and maternal genetic structure of the species.

### 3.7. Study Limitations and Future Perspectives

Our sampling was unbalanced across the species’ range, with population sample sizes ranging from one individual (Kodar) to 39 (Kharaulakh). This imbalance is largely explained by objective factors: some populations inhabit remote and difficult-to-access mountain regions, while others are listed in regional or national Red Data Books, which limited our ability to collect larger samples. Consequently, differences in haplogroup frequencies among populations with small sample sizes should be interpreted with caution. Furthermore, the apparent geographic restriction of some lineages and the absence of rare haplogroups or sub-haplogroups from particular populations may partly reflect limited sampling rather than true geographic absence. For instance, only sub-haplogroup C3a was detected in the Stanovoy population (*n* = 5), and only B1 in the Putorana population (*n* = 6); given these small sample sizes, the presence of additional rare lineages in these regions cannot be excluded. The reliability of diagnostic positions for lineages represented by few specimens, such as sub-haplogroup C5 (represented by a single Kodar individual), may also be limited. Future collection of additional samples from underrepresented populations will help refine the diagnostic positions specific to particular haplogroups and sub-haplogroups. Nevertheless, given the large overall sample size (*n* = 257) and the wide geographic coverage encompassing all major mountain systems, the overall hierarchical structure of the mitochondrial haplogroups identified in this study is well supported and is unlikely to be substantially altered by the addition of new data.

## 4. Conclusions

Analysis of 257 complete mitochondrial genomes of *O. nivicola* revealed a pronounced phylogeographic structure across the species’ range and enabled the identification of four major mitochondrial haplogroups (A–D) with multiple nested sub-haplogroups.

Haplogroup A was the most divergent lineage and was largely restricted to the Kamchatka Peninsula and a small part of the Koryak Mountains, suggesting long-term isolation of these populations. Haplogroup B was predominantly associated with northern populations and showed a fragmented distribution, suggesting the persistence of ancient mitochondrial lineages in regional refugia. Haplogroup C was the most widespread and genetically diverse lineage, spanning a large part of the species distribution range and likely preserving a substantial portion of the ancestral mitochondrial diversity of snow sheep. Haplogroup D exhibited a restricted distribution centered in the Verkhoyansk region, reflecting localized evolutionary differentiation.

The identification of numerous haplogroup- and sub-haplogroup-specific diagnostic mutations provides a robust framework for future studies on snow sheep phylogeography, population history, conservation genetics, and taxonomy. The observed patterns of mitochondrial diversity highlight the major role of Pleistocene climatic oscillations and long-term geographic isolation in shaping the current genetic structure of snow sheep populations.

Overall, this study substantially expands our knowledge of mitochondrial diversity in *O. nivicola* and establishes the first comprehensive mitochondrial haplogroup system for the species. These findings provide a foundation for future integrative research combining mitochondrial, nuclear genomic, morphological, ecological, and paleontological data to further elucidate the evolutionary history and inform conservation strategies for this unique mountain ungulate. In particular, integrating genetic data with information on past climatic changes, geological history, and fossil records will be important for identifying potential Pleistocene refugia, reconstructing historical population dynamics, and better understanding the factors that shaped the current phylogeographic structure of snow sheep.

## Figures and Tables

**Figure 1:**
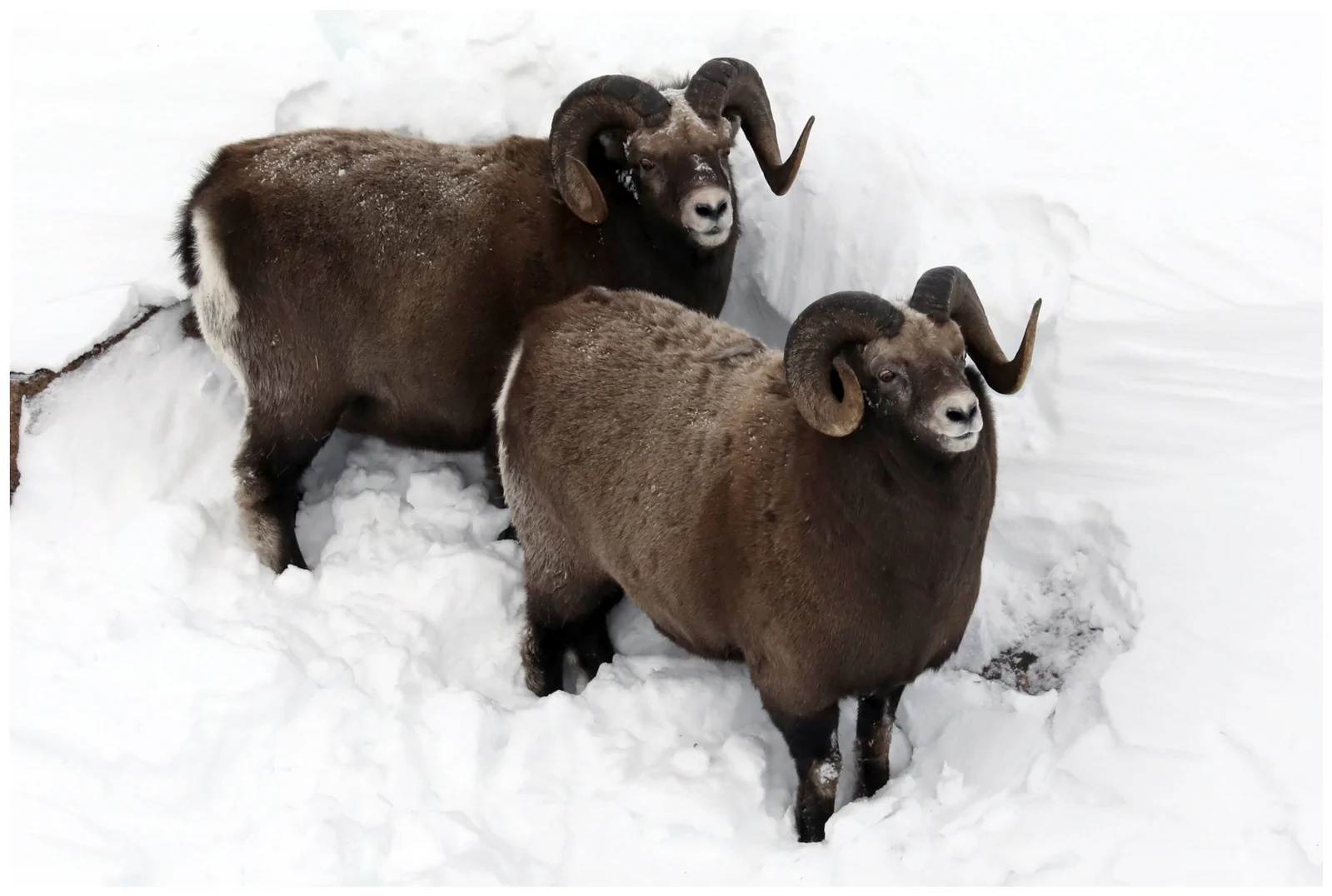
Representative individuals of *Ovis nivicola* from the Putorana Plateau population. Author’s photograph by Mikhail G. Bondar.

**Figure 2:**
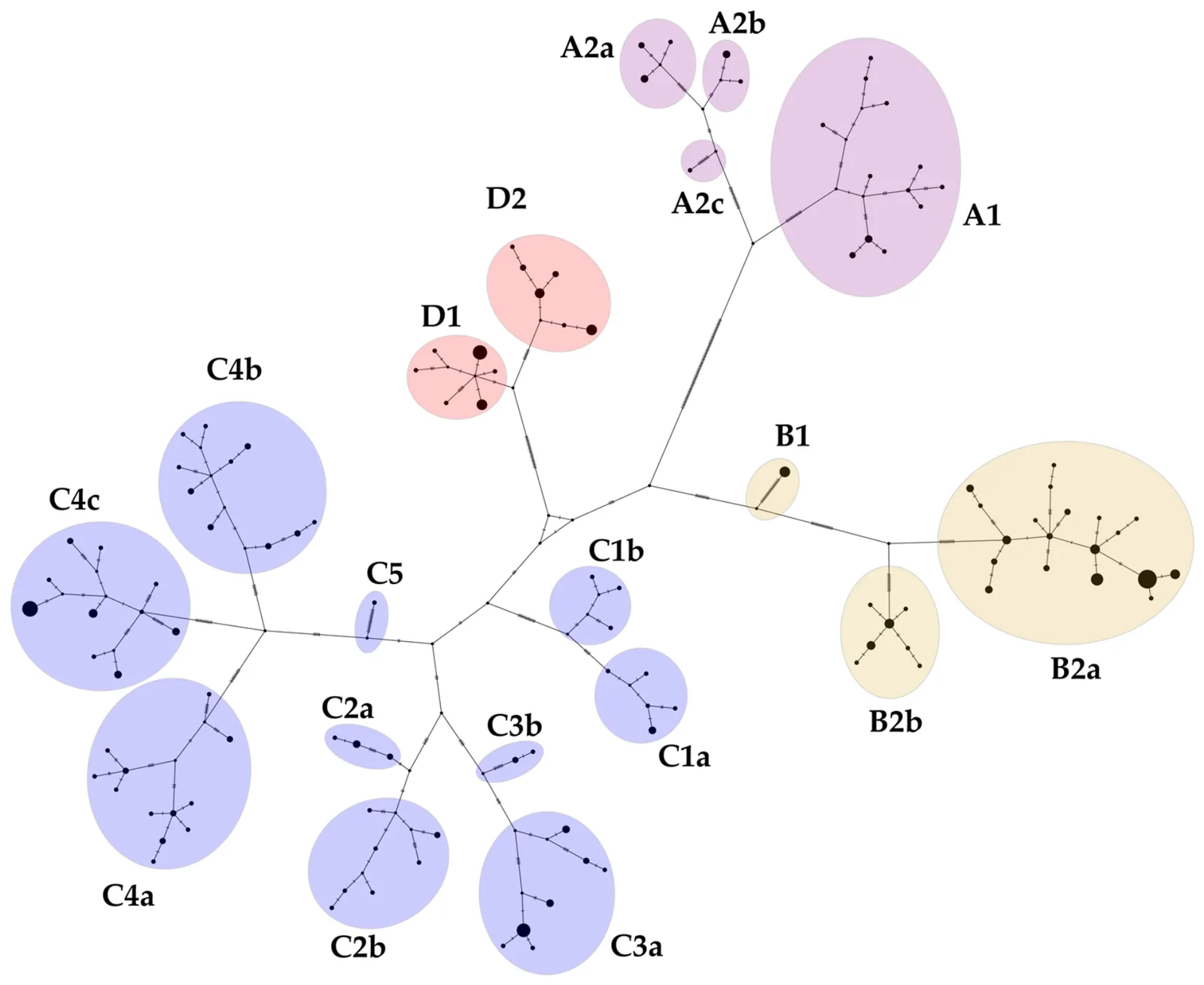
Median-joining network of *Ovis nivicola* mitochondrial sequences (*n* = 257) based on the 13 protein-coding genes and two rRNA genes. Black circles represent haplotypes; circle size is proportional to the number of individuals. Haplotypes belonging to the major haplogroups (A–D) and their sub-haplogroups are outlined in color. Hatch marks on the branches indicate the number of mutational steps.

**Figure 3:**
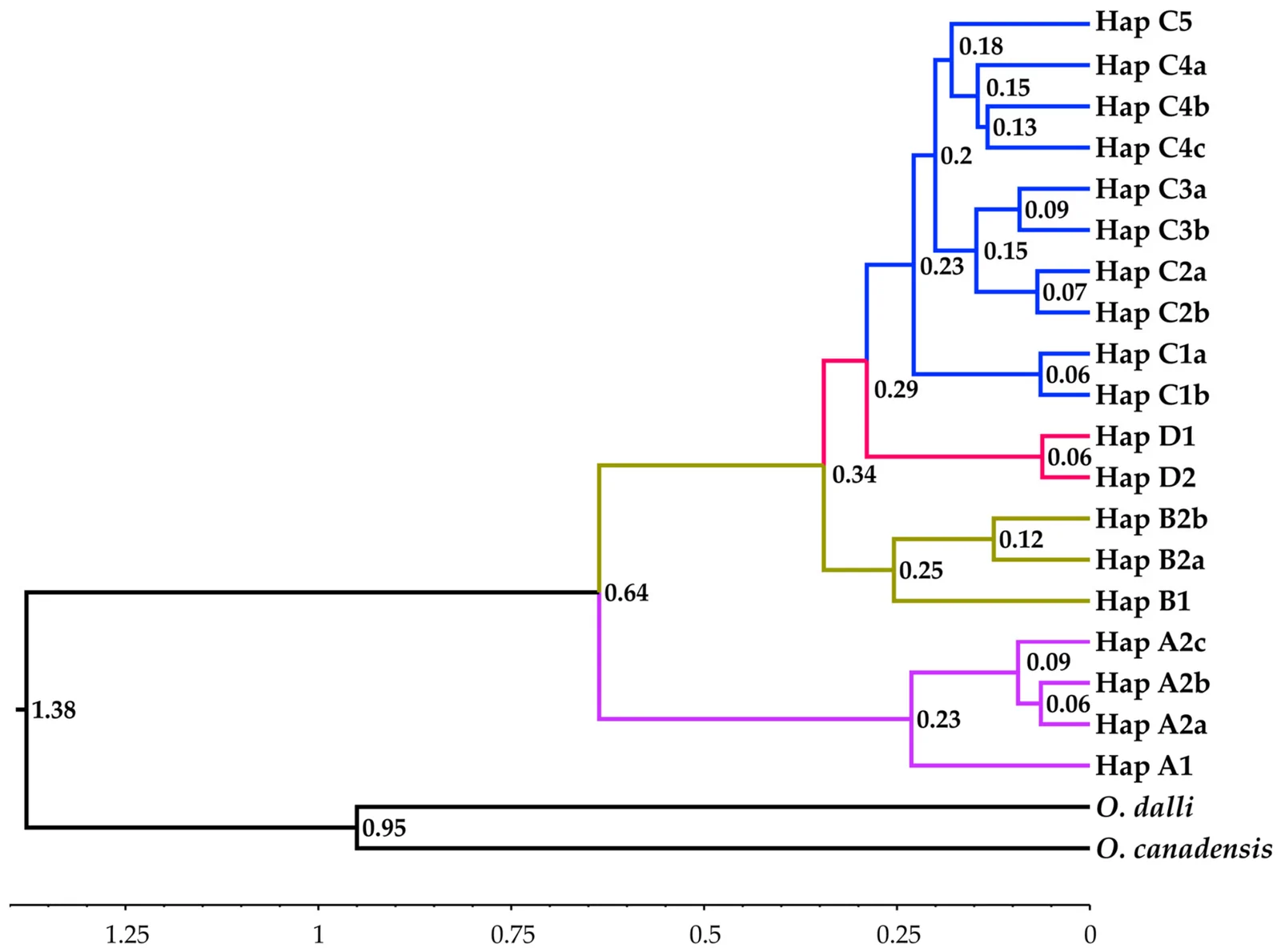
Bayesian phylogenetic tree of mitochondrial haplogroups in *Ovis nivicola* inferred from the concatenated alignment of 13 protein-coding genes and two rRNA genes (13,932 bp). Branches are colored according to mitochondrial haplogroups: haplogroup A (magenta), haplogroup B (olive), haplogroup C (blue), and haplogroup D (red). Divergence times are shown in million years ago. *O. canadensis* and *O. dalli* were used as outgroups. The split between *O. canadensis* and *O. dalli* (~950 kya) was used as a fixed calibration point according to Rezaei et al. [43]. Posterior probability values were 1 for all the nodes except for the node separating sub-haplogroups C4b and C4c (posterior probability = 0.89). The 95% highest posterior density (HPD) intervals for the estimated divergence times are presented in the supplementary phylogenetic tree (Supplementary Figure S1).

**Figure 4:**
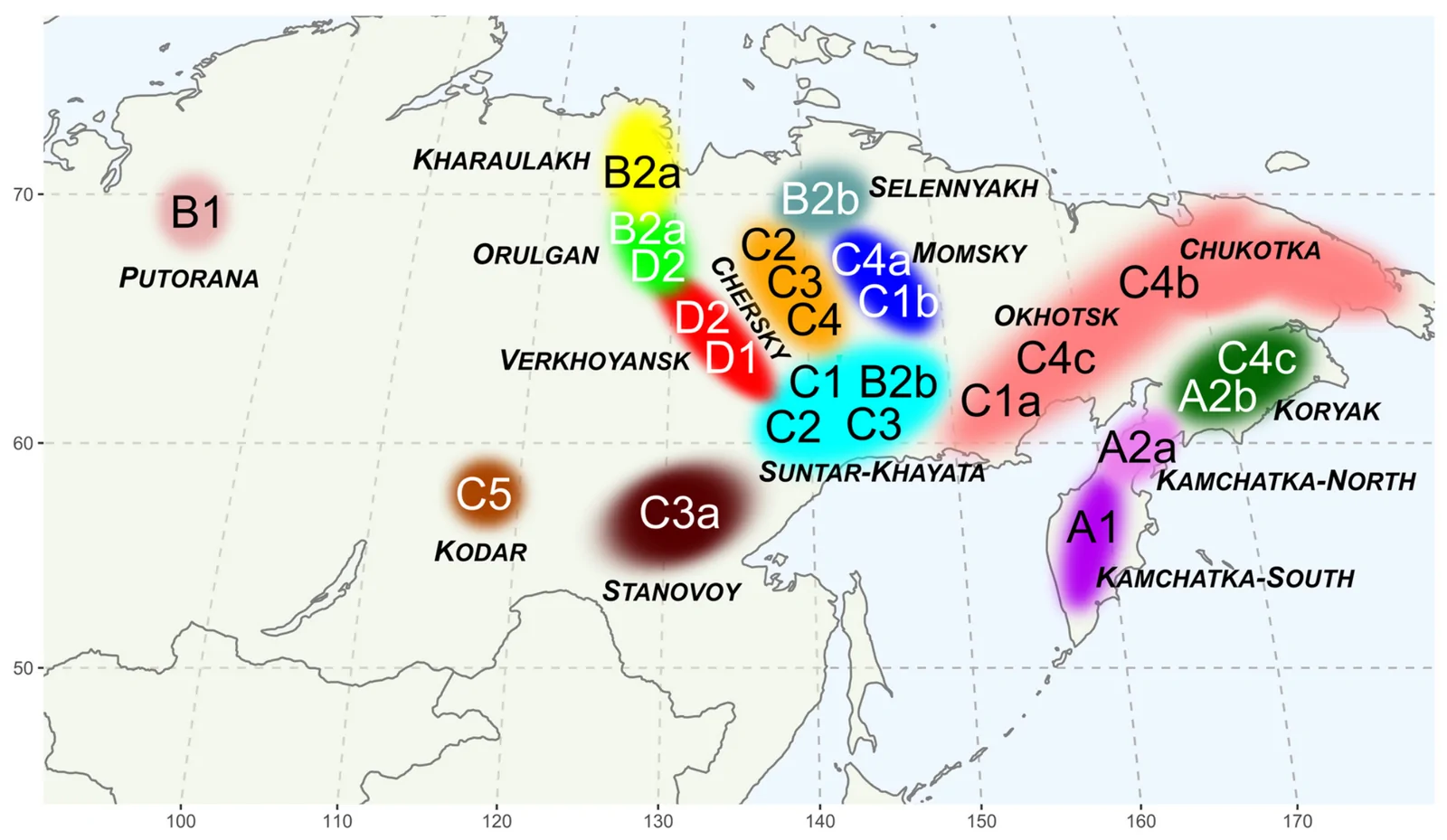
Geographic distribution of mitochondrial haplogroups and sub-haplogroups of *Ovis nivicola*. Populations are shown in different colors, with the corresponding mitochondrial haplogroups and sub-haplogroups indicated within each population. The geographic areas shown are schematic representations based on the sampling locations and are not intended to indicate precisely delimited geographic ranges.

**Table 1:** Geographic origin, population assignment, sample size, and data source for *Ovis nivicola* specimens used in this study.

Location/Mountain Range	Population	*n*	Source
Southern Sredinny Ridge; Eastern Range (Kamchatka)	Kamchatka south	14	[23], this study
Northern Sredinny Ridge (Kamchatka)	Kamchatka north	8	this study
Koryak Mountains	Koryak	31	[23], this study
Anadyr Highlands; Chukotka Mountains	Chukotka	6	[23], this study
Kolyma Mountains	Okhotsk	13	[23], this study
Moma Range, Yakutia	Momsky	12	[23], this study
Chersky Range, Yakutia	Chersky	19	[15,23], this study
Selennyakh Range, Yakutia	Selennyakh	9	this study
Central Verkhoyansk Range, Yakutia	Verkhoyansk	32	this study
Orulgan Range, Yakutia	Orulgan	30	this study
Kharaulakh Range, Yakutia	Kharaulakh	39	this study
Suntar-Khayata Range, Yakutia	Suntar-Khayata	32	[23], this study
Stanovoy Range	Stanovoy	5	this study
Kodar Mountains	Kodar	1	this study
Putorana Plateau	Putorana	6	this study

**Table 2:** Geographic composition of mitochondrial haplogroups and sub-haplogroups identified in *Ovis nivicola*.

Haplogroup	Sub-Haplogroup	Population(s) and Sample Size
A	A1	Kamchatka South (*n* = 14), Kamchatka North (*n* = 1)
	A2a	Kamchatka North (*n* = 6)
	A2b	Koryak (*n* = 4)
	A2c	Kamchatka North (*n* = 1)
B	B1	Putorana (*n* = 6)
	B2a	Kharaulakh (*n* = 39), Orulgan (*n* = 24)
	B2b	Selennyakh (*n* = 8), Suntar-Khayata (*n* = 6)
C	C1a	Suntar-Khayata (*n* = 5), Okhotsk (*n* = 2)
	C1b	Momsky (*n* = 3)
	C2a	Suntar-Khayata (*n* = 4), Chersky (*n* = 2)
	C2b	Chersky (*n* = 7), Selennyakh (*n* = 1)
	C3a	Suntar-Khayata (*n* = 15), Stanovoy (*n* = 5), Okhotsk (*n* = 1)
	C3b	Suntar-Khayata (*n* = 2), Chersky (*n* = 1)
	C4a	Momsky (*n* = 9), Chersky (*n* = 7)
	C4b	Okhotsk (*n* = 8), Chukotka (*n* = 5), Chersky (*n* = 2)
	C4c	Koryak (*n* = 27), Okhotsk (*n* = 2), Chukotka (*n* = 1)
	C5	Kodar (*n* = 1)
D	D1	Verkhoyansk (*n* = 21)
	D2	Verkhoyansk (*n* = 11), Orulgan (*n* = 6)

## Data Availability

The complete mitochondrial genome sequences generated in this study have been deposited in the NCBI GenBank database under accession numbers QB006217–QB006446.

## Supplementary Materials

The following supporting information can be downloaded at https://www.mdpi.com/article/10.3390/ani16193018/s1, Figure S1: Bayesian phylogenetic tree of mitochondrial haplogroups in *Ovis nivicola* indicating 95% highest posterior density (HPD) intervals for divergence times. The tree topology is identical to Figure 3, but node labels show the 95% HPD intervals for divergence times instead of point estimates. *Ovis canadensis* and *Ovis dalli* were used as outgroups. The split between *O. canadensis* and *O. dalli* (~950 kya) was used as a calibration point according to Rezaei et al. [43]. Posterior probability values were 1 for all nodes except the node separating sub-haplogroups C4b and C4c (posterior probability = 0.89); Table S1: PartitionFinder2 results for the analysis using one representative sequence per haplogroup/sub-haplogroup; Table S2: Complete list of diagnostic mutations; Table S3: Number of diagnostic mutations by haplogroup.

## Abbreviations

The following abbreviations are used in this manuscript:

mtDNA	Mitochondrial DNA
SNPs	Single nucleotide polymorphisms
kya	Thousand years ago
NCBI	National Center for Biotechnology Information
