# Supplementary material for: Comprehensive Identification and Description of Mitochondrial DNA Haplogroups and Sub-Haplogroups in Snow Sheep (Ovis nivicola) in Support of Their Evolutionary History and Conservation
Source: Animals (Basel). 2026 Sep 25;16(19):3018. doi: 10.3390/ani16193018 (PMC13663522; doi:10.3390/ani16193018)
Supplement: Supplementary file 1 [file animals-16-03018-s001.zip › animals-4528453-supplementary.pdf]

Supplementary Materials

Comprehensive Identification and Description of Mitochondrial DNA Haplogroups and Sub-haplogroups in Snow Sheep (*Ovis nivicola*) in Support of Their Evolutionary History and Conservation

Arsen V. Dotsev, Innokentiy M. Okhlopkov, Olga A. Koshkina, Mikhail G. Bondar, Dennis I. Litovka, Andrey A. Sitsko, Taras P. Sipko, Neckruz F. Bakoev, Nikolay V. Mamaev, Veronika R. Kharzinova, Tatiana E. Deniskova, Maria N. Semerikova, Vugar A. Bagirov, Michael N. Romanov, Darren K. Griffin, Ivica Medugorac, Gottfried Brem and Natalia A. Zinovieva

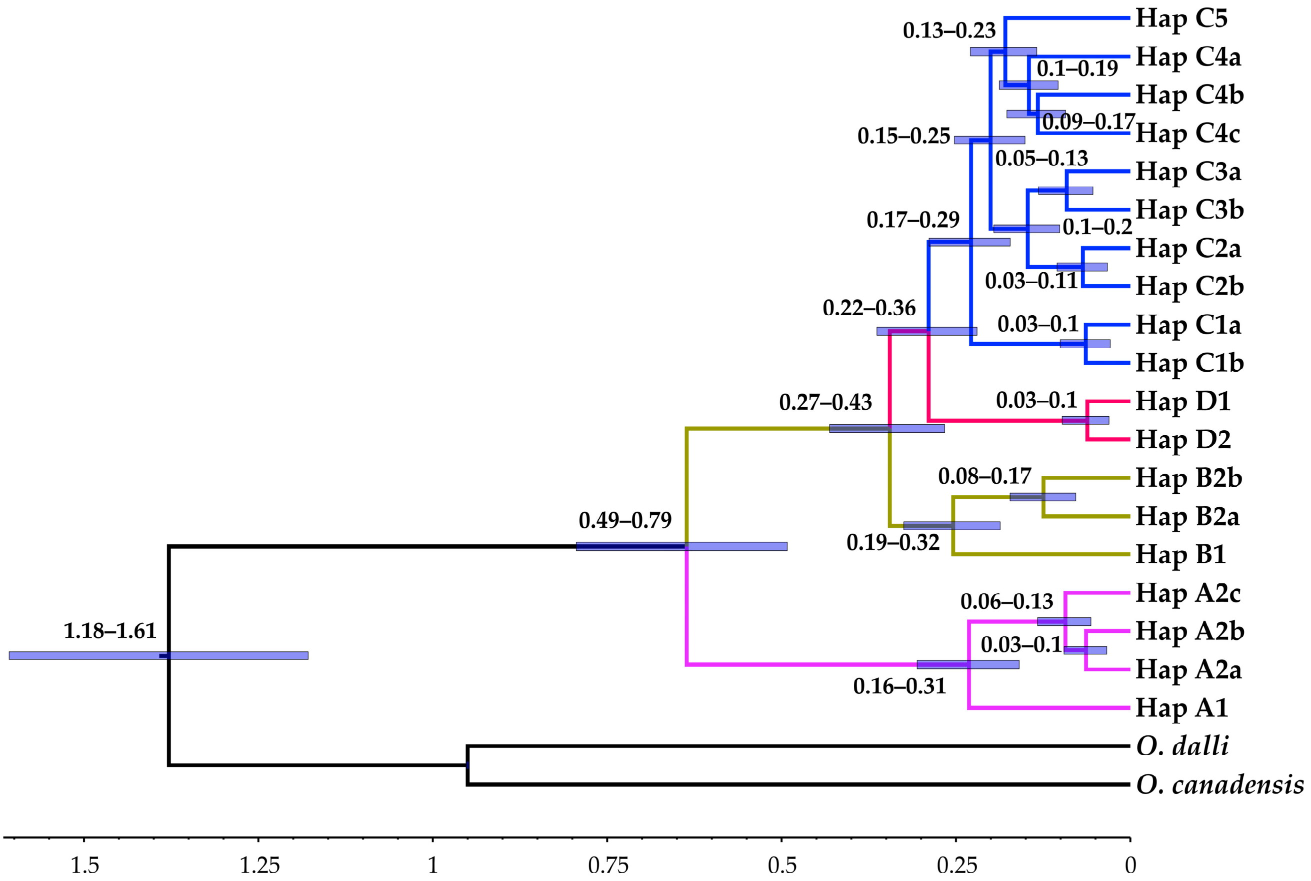

Supplementary Figure S1. Bayesian phylogenetic tree of mitochondrial haplogroups in *Ovis nivicola* indicating 95% highest posterior density (HPD) intervals for divergence times. The tree topology is identical to Figure 3, but node labels show the 95% HPD intervals for divergence times instead of point estimates. *Ovis canadensis* and *Ovis dalli* were used as outgroups. The split between *O. canadensis* and *O. dalli* (~950 kya) was used as a calibration point according to Rezaei et al. [43]. Posterior probability values were 1 for all nodes except the node separating sub-haplogroups C4b and C4c (posterior probability = 0.89).

Supplementary Table S1. PartitionFinder2 results for the analysis using one representative sequence per haplogroup/sub-haplogroup.

Subset	Best Model	# sites	Partition names
1	GTR+G+X	3131	nd5_pos1, rna16S, rna12S
2	TRN+X	663	nd3_pos1, cox2_pos1, nd1_pos1
3	TRN+X	1109	nd1_pos2, nd2_pos2, nd4l_pos2, atp6_pos2, nd3_pos2
4	HKY+I+X	1047	cytb_pos3, nd2_pos3, nd1_pos3
5	TRN+X	414	atp8_pos3, nd2_pos1
6	TRN	515	cox1_pos1
7	TRN+X	1123	cox2_pos2, cox1_pos2, cytb_pos2
8	HKY+X	743	cox2_pos3, cox1_pos3
9	HKY+X	132	atp8_pos1, atp8_pos2
10	HKY+X	687	nd4_pos1, atp6_pos1
11	TRN+X	343	atp6_pos3, nd3_pos3
12	K80	262	cox3_pos1
13	HKY+I	1330	cox3_pos2, nd5_pos2, nd4_pos2
14	TRN+X	871	nd5_pos3, cox3_pos3
15	HKY+I	479	nd4l_pos1, cytb_pos1
16	HKY+X	558	nd4l_pos3, nd4_pos3
17	HKY+X	175	nd6_pos1
18	HKY+X	175	nd6_pos2
19	HKY	175	nd6_pos3

Abbreviations: TRN, Tamura–Nei model; HKY, Hasegawa–Kishino–Yano model; K80, Kimura 2-parameter model; I, invariant sites; G, gamma-distributed rate variation; X, empirical base frequencies.

Supplementary Table S2. Complete list of diagnostic mutations

Group	Position	Ref_nt	Alt_nt	Specificity
A	809	C	T	100%
A	969	T	C	100%
A	1485	T	C	100%
A	1504	C	T	100%
A	1894	C	A	100%
A	2146	A	G	100%
A	2148	T	C	100%
A	3150	G	A	100%
A	3168	T	C	100%
A	3345	T	C	100%
A	3393	C	T	100%
A	3488	G	A	100%
A	4018	C	T	100%
A	4298	C	T	100%
A	4498	C	T	100%
A	4531	C	T	100%
A	4881	C	T	100%
A	5440	A	T	100%
A	5650	C	T	100%
A	5734	T	C	100%
A	6058	A	G	100%
A	6742	T	C	100%
A	7139	T	C	100%
A	7232	T	C	100%
A	7250	T	C	100%
A	7319	G	A	100%
A	7449	C	T	100%

A	7595	C	T	100%
A	7754	A	G	100%
A	7995	C	T	100%
A	8113	C	T	100%
A	8464	G	A	100%
A	8506	T	C	100%
A	8539	C	T	100%
A	8703	T	C	100%
A	9189	A	G	100%
A	9219	G	A	100%
A	9246	T	C	100%
A	9664	C	T	100%
A	9742	C	T	100%
A	10143	C	T	100%
A	10493	A	G	100%
A	10676	C	T	100%
A	11027	G	A	100%
A	11186	T	C	100%
A	11430	C	T	100%
A	11451	G	A	100%
A	11525	T	C	100%
A	11759	C	T	100%
A	11768	T	C	100%
A	11866	T	C	100%
A	12077	A	G	100%
A	12233	G	A	100%
A	12269	C	T	100%
A	12476	G	A	100%

A	12846	G	A	100%
A	13165	A	G	100%
A	13753	C	T	100%
A	13785	T	A	100%
A	14236	T	C	100%
A	14479	C	T	100%
A	14626	T	C	100%
A	14827	T	C	100%
A	14899	T	C	100%
A	15139	T	C	100%
A	15350	G	A	100%
A	15519	C	T	100%
A	15565	A	-,C	100%
A	15644	G	A	100%
A	15846	G	A	100%
A	15973	C	T	100%
A	16017	T	C	100%
A	16050	C	T	100%
A	16404	T	C	100%
A1	1130	C	T	100%
A1	4786	C	T	100%
A1	5404	T	C	100%
A1	5866	A	G	100%
A1	6205	C	T	100%
A1	10002	T	C	100%
A1	10556	T	C	100%
A1	11348	T	C	100%
A1	11487	G	T	100%

A1	13064	C	T	100%
A1	13514	C	T	100%
A1	13557	T	C	100%
A1	15785	G	A	100%
A1	15877	C	T	100%
A2	2206	C	T	100%
A2	3549	C	T	100%
A2	7080	T	C	100%
A2	7295	T	C	100%
A2	8155	A	G	100%
A2	8356	G	A	100%
A2	8838	C	T	100%
A2	9541	C	T	100%
A2	10044	T	C	100%
A2	10734	T	C	100%
A2	11105	A	G	100%
A2	13091	T	C	100%
A2	13124	G	A	100%
A2	14977	C	T	100%
A2a	291	C	T	100%
A2a	637	C	T	100%
A2a	2889	T	C	100%
A2a	8922	T	C	100%
A2a	9921	T	C	100%
A2a	10506	T	A	100%
A2a	13951	T	C	100%
A2a	14357	G	A	100%
A2b	6655	C	T	100%

A2b	10838	G	A	100%
A2b	11069	C	T	100%
A2b	15561	A	G	100%
A2c	1720	C	T	100%
A2c	2916	T	C	100%
A2c	5521	T	C	100%
A2c	5696	A	G	100%
A2c	6340	C	T	100%
A2c	8270	A	G	100%
A2c	10340	C	T	100%
A2c	15662	C	T	100%
A2c	15850	A	G	100%
B	1662	T	C	100%
B	7580	C	T	100%
B	7671	C	T	100%
B	8068	T	C	100%
B	9879	T	C	100%
B	10238	C	T	100%
B	11885	T	C	100%
B	12437	T	C	100%
B	14438	T	C	100%
B	15627	T	C	100%
B	15997	G	A	100%
B1	105	C	T	100%
B1	535	C	T	100%
B1	956	A	-,T	100%
B1	1200	T	C	100%
B1	2637	C	T	100%

B1	2775	C	T	100%
B1	3375	T	C	100%
B1	3777	C	T	100%
B1	5897	T	C	100%
B1	6493	C	T	100%
B1	9604	A	G	100%
B1	10457	T	C	100%
B1	10865	T	C	100%
B1	11878	A	G	100%
B1	13232	T	C	100%
B1	13509	T	C	100%
B1	13912	C	T	100%
B1	14337	T	C	100%
B1	15118	T	C	100%
B1	15238	T	C	100%
B1	15314	T	C	100%
B1	15540	C	T	100%
B1	15674	G	A	100%
B1	15812	G	A	100%
B2	1682	C	T	100%
B2	2793	C	T	100%
B2	3114	T	C	100%
B2	3243	C	T	100%
B2	4619	A	G	100%
B2	5545	A	G	100%
B2	8536	G	A	100%
B2	11381	T	C	100%
B2	11477	C	T	100%

B2	12656	C	T	100%
B2	13415	T	C	100%
B2	13484	T	C	100%
B2	14470	C	T	100%
B2	14680	T	C	100%
B2	15610	C	T	100%
B2a	334	A	G	100%
B2a	428	A	G	100%
B2a	1815	A	G	100%
B2a	2731	T	C	100%
B2a	4039	C	T	100%
B2a	5722	C	T	100%
B2a	9159	C	T	100%
B2a	9721	T	A	100%
B2a	12579	G	A	100%
B2a	13359	T	C	100%
B2b	1144	C	T	100%
B2b	2392	T	C	100%
B2b	7091	T	C	100%
B2b	7721	T	C	100%
B2b	9192	C	T	100%
B2b	9822	C	T	100%
B2b	10062	A	G	100%
B2b	11903	T	C	100%
B2b	11963	T	C	100%
B2b	12101	G	A	100%
B2b	12801	T	C	100%
B2b	13585	T	C	100%

B2b	14383	C	T	100%
B2b	15986	G	A	100%
C	17	T	C	100%
C	733	T	A,C	100%
C	4115	T	C	100%
C	11832	T	C	100%
C	13106	C	T	100%
C1	3949	C	A,T	100%
C1	6247	T	C	100%
C1	8781	C	T	100%
C1	11294	C	T	100%
C1	12137	G	A	100%
C1	14032	T	C	100%
C1	14467	C	T	100%
C1	14662	T	C	100%
C1	14890	G	A	100%
C1	16388	C	T	100%
C1a	826	C	T	100%
C1a	3390	C	T	100%
C1a	3934	C	T	100%
C1a	4030	C	T	100%
C1a	5767	G	A	100%
C1a	15638	C	T	100%
C1a	15988	C	T	100%
C1b	102	T	C	100%
C1b	1087	G	A	100%
C1b	14953	T	C	100%
C1b	15863	G	A	100%

C2	2962	C	T	100%
C2	6791	T	C	100%
C2	14629	C	T	100%
C2	15635	G	A	100%
C2a	10190	C	T	100%
C2a	14563	G	A	100%
C2b	9610	C	T	100%
C2b	14887	T	A	100%
C2b	15719	G	A	100%
C3	2190	T	C	100%
C3	3432	C	T	100%
C3	3947	C	G	100%
C3	10820	G	A	100%
C3	14677	C	T	100%
C3	15358	G	A	100%
C3a	724	C	T	100%
C3a	3927	C	T	100%
C3a	7115	C	T	100%
C3a	14875	C	T	100%
C3a	15918	A	G	100%
C3b	3261	G	A	100%
C3b	3938	T	C	100%
C3b	4432	A	G	100%
C4	4339	T	C	100%
C4	10170	C	T	100%
C4	12209	T	C	100%
C4	12470	T	C	100%
C4	13340	C	T	100%

C4a	456	C	T	100%
C4a	1816	C	T	100%
C4a	2904	C	T	100%
C4a	4517	C	T	100%
C4a	7649	C	T	100%
C4a	7866	T	C	100%
C4a	10554	A	G	100%
C4a	10601	T	C	100%
C4a	11358	T	C	100%
C4b	864	C	T	100%
C4b	3916	C	T	100%
C4b	5446	T	C	100%
C4b	5647	C	T	100%
C4b	6061	G	A	100%
C4b	10080	C	T	100%
C4b	14656	A	G	100%
C4c	734	C	T	100%
C4c	5566	T	C	100%
C4c	8017	C	T	100%
C4c	9772	T	C	100%
C4c	10877	T	C	100%
C4c	11327	G	A	100%
C4c	11432	T	C	100%
C4c	12572	C	T	100%
C4c	13292	T	C	100%
C4c	13776	T	C	100%
C4c	14747	C	T	100%
C4c	15228	C	T	100%

C5	1368	A	G	100%
C5	2910	T	C	100%
C5	4522	T	C	100%
C5	4675	T	C	100%
C5	6046	C	T	100%
C5	9028	T	C	100%
C5	9252	T	C	100%
C5	9994	G	A	100%
C5	11455	C	T	100%
C5	11508	A	C	100%
C5	11846	C	T	100%
C5	13406	C	T	100%
C5	13588	C	T	100%
C5	14695	C	T	100%
C5	15169	G	A	100%
C5	15316	G	A	100%
C5	15738	G	A	100%
C5	15776	C	A,G	100%
C5	15822	G	A	100%
C5	15836	C	T	100%
C5	16272	T	C	100%
D	538	A	G	100%
D	733	C	A,T	100%
D	935	C	T	100%
D	1244	G	A	100%
D	1673	A	G	100%
D	3324	T	A	100%
D	3919	G	A	100%

D	4144	A	G	100%
D	4490	T	C	100%
D	4699	A	G	100%
D	5936	C	T	100%
D	6473	C	T	100%
D	7316	T	C	100%
D	8024	T	C	100%
D	8491	C	T	100%
D	9117	T	C	100%
D	10283	C	T	100%
D	10750	A	G	100%
D	11900	T	C	100%
D	13552	T	C	100%
D	15488	T	C	100%
D	15619	A	G	100%
D	16213	G	A	100%
D1	14221	T	C	100%
D1	15609	G	T	100%
D2	1082	C	T	100%
D2	3540	A	G	100%
D2	4549	C	T	100%
D2	6031	T	C	100%
D2	6731	T	C	100%
D2	7167	C	T	100%
D2	10532	C	T	100%
D2	12344	C	T	100%
D2	15708	C	T	100%
D2	15920	T	C	100%

Note: Ref_nt, nucleotide in the reference mitochondrial genome (NC_039431.1); Alt_nt, alternative nucleotide(s) defining the given haplogroup/sub-haplogroup. "–" denotes a deletion; "A,C" or similar indicates that more than one alternative variant was found within the group.

Supplementary Table S3. Number of diagnostic mutations by haplogroup

Haplogroup / Sub-haplogroup	Number of diagnostic mutations
A	74
A1	14
A2	14
A2a	8
A2b	4
A2c	9
B	11
B1	24
B2	15
B2a	10
B2b	14
C	5
C1	10
C1a	7
C1b	4
C2	4
C2a	2
C2b	3
C3	6
C3a	5
C3b	3
C4	5
C4a	9

C4b	7
C4c	12
C5	21
D	23
D1	2
D2	10
